# An Integrated, CFD-Based, Analysis of Carbonation in a Stirred Tank Reactor

**DOI:** 10.3390/ma18071535

**Published:** 2025-03-28

**Authors:** Georgios P. Gakis, Danai Marinos, Ioannis G. Aviziotis, Efthymios Balomenos, Andreas G. Boudouvis, Dimitrios Panias

**Affiliations:** 1 School of Chemical Engineering, National Technical University of Athens (NTUA), Heroon Polytechniou 9, 15780 Zografou, Greece; javiziot@chemeng.ntua.gr (I.G.A.); boudouvi@chemeng.ntua.gr (A.G.B.); 2 Laboratory of Metallurgy, School of Mining and Metallurgical Engineering, National Technical University of Athens (NTUA), Heroon Polytechniou 9, 15780 Zografou, Greece; dmarinou@metal.ntua.gr (D.M.); thymis@metal.ntua.gr (E.B.); panias@metal.ntua.gr (D.P.)

**Keywords:** carbonation, CFD modelling, multiphase flow, transport phenomena, stirred tank

## Abstract

Carbonation precipitation processes have been widely used due to their numerous applications in a wide range of fields. The complexity of these processes lies within the interplay of transport phenomena, multiphase flows, chemical reactions, and solid precipitation, deeming the experimental analysis and in-depth mechanistic understanding of the process dynamics a rather challenging task. In this work, a three-dimensional CFD model is developed, focusing on the carbonation step of the carbonation precipitation process, taking into account the flow dynamics of the liquid solution in the stirred tank, the CO_2_ bubble flow, and the dissolution in the liquid solution, as well as its dissociation in water. The model is validated with experimental measurements, and a very good agreement is achieved. Additionally, a parametric analysis is conducted to study the effect of different process parameters, such as temperature, CO_2_ flow rate, and rotational speed. The analysis of the different phenomena and their interplay reveals the key mechanisms that dictate the carbonation step, resulting in an in-depth understanding of the process. The presented computational approach can potentially pave the way towards a knowledge-based process and reactor design; thus, assisting the scale-up of such processes in stirred tank reactors.

## 1. Introduction

Carbonation is a process where CO_2_ gas reacts with a solid or solution to form carbonate species or carbonic acid, bicarbonate, and carbonate ions, respectively. This process is seen in many different fields, including beverage production, geology, carbon capture, and precipitation. In geology, scientists study the reactions between the atmosphere and the ores, especially for CO_2_ sequestration, where, for example, wollastonite can react with CO_2_ gas to form calcite and quartz, which occurs naturally in nature [1,2]. Carbonation has also been applied by introducing carbon dioxide to alkaline solutions to capture the CO_2_ known as chemical absorption [3]. In this process, caustic solutions such as NaOH, KOH, etc., have higher absorption efficiency compared to amine and ammonia solutions [4]. Lastly, the carbonation process can find application in precipitation processes where CO_2_ is added to alkaline solutions to precipitate a solid compound by lowering the pH.

The most long-established process of carbonation precipitation is the sintering and lime-sintering process to produce alumina [5,6]. In both processes, aluminosilicate minerals such as bauxites, nepheline syenites, and anorthosites are mixed with sodium carbonate and/or limestone and sintered at high temperatures to produce sodium aluminates and calcium silicates. The produced slag is leached with water or sodium carbonate to dissolve sodium aluminate while calcium silicate is not dissolved. To precipitate alumina hydrates from this solution, carbonation is applied, leaving, after filtration, a sodium carbonate solution that can be recycled back to the leaching step [7].

In the carbonation precipitation, carbon dioxide gas is added to the alkaline sodium aluminate solution. Firstly, gaseous CO_2_ transforms into aqueous CO_2_ according to reaction (a1). Aqueous CO_2_ is hydrolyzed according to reactions (a2) and (a3), therefore gradually neutralizing the alkaline solution, leading to the dissociation of the aluminate ions according to reaction (a4), precipitating aluminum trihydrate.

CO_2(g)_ ↔ CO_2(aq)_ (a1)

CO_2(aq)_ + H_2_O_(l)_ ↔ HCO_3_^−^_(aq)_ + H^+^_(aq)_ (a2)

HCO_3_^−^_(aq)_ ↔ CO_3_^−2^_(aq)_ + H^+^_(aq)_ (a3)

Al(OH)_4_^−^_(aq)_ ↔ Al(OH)_3(s)_ + OH^−^_(aq)_ (a4)

Carbonation precipitation has been studied extensively by many authors to recover Al. It has been shown that the thermodynamically stable alumina hydrate phase precipitated during the carbonation is bayerite [8,9,10,11], a polymorph of gibbsite, which is the product of the Bayer process. The process can become even more complex, as has been shown in previous works of the authors [8,9], extensive carbonation or high initial sodium carbonate content in the solution can lead to a phase named dawsonite, NaAlCO_3_(OH)_2_, which is unwanted if metallurgical-grade alumina is the target product.

The complexity [9,12] of the described process led to the need to model the carbonation process. The process is driven by the interplay between different transport phenomena, as well as physicochemical mechanisms and chemical reactions [13]. The combination of CO_2_ gas flow in the form of bubbles in the liquid solution [14], the CO_2_ dissolution, as well as the transport and reactions of diluted species in the solution, result in a two-phase flow within the carbonation precipitation reactor [15,16,17], excluding, in the first place, the solid phases from the precipitation process. As such processes usually take place in stirred tank reactors [8], the rotation dynamics influence all the above mechanisms. The aforementioned phenomena are coupled, with the overall process dynamics and products being dictated by their interplay [12,18]. Furthermore, these phenomena occur simultaneously but at different time scales due to the corresponding kinetics, which may vary significantly. Thus, the in-depth understanding of the individual mechanisms, along with their impact on the final product, is a non-trivial task, since the identification of limiting and dominating mechanisms and the extraction of mechanistic insight are highly challenging for conventional experimental measurement techniques [12,19].

In this context, computational modelling has emerged as an invaluable tool to study and analyze such processes. Miao et al. have developed a kinetic model for aqueous mineral carbonation in stirred reactors [20], while Mitchell et al. developed a kinetic model for mineral carbonation in the ocean [21]. Computational Fluid Dynamics (CFD) modeling has been incorporated by Brucato et al. for the simulation of stirred tanks [22]. Over the years, several works have used CFD to study the liquid-solid flow and precipitation within stirred tank reactors, as in the works of Zhao et al. [23,24]. Moguel et al. used CFD to study barium carbonate precipitation in fluidized bed reactors [25]. Barium sulfate precipitation has also been studied using CFD models by Wang et al. [26] and Cheng et al. [27], while potassium carbonation has been studied by Yu et al. [28].

In recent years, CFD models have been successfully developed for the design of stirred tanks for mixing [29] as well as precipitation [30]. Furthermore, CFD models concerning the gas bubble transport within stirred tanks have been developed by Kim et al. [13,31] and Hu et al. offering insights into impeller design [32]. Gas-liquid interactions have been studied by CFD in composites [33], while the combination of CFD with population balance model has been used by Li et al. [34] and Chen et al. [35]. Although some of these models include the CO_2_ dissolution and dissociation within the tank, the results regarding the CO_2_ dissociation and the effect on the pH of the solution lack experimental validation. The development of models that incorporate a more complete range of phenomena has proceeded but with less focus on mechanistic insights [13,17,36]. Finally, combined machine learning and CFD approaches have been developed very recently [37], with more focus on the predictive capabilities of the model. In any case, the mechanistic insight regarding the interplay between mechanisms that govern the process, as well as its effect on key properties of the solution such as the pH, has not been addressed in detail. Furthermore, the effect of key process parameters, such as temperature and inflow rates, on the overall carbonation process and the constituting physicochemical mechanisms is not studied in detail by CFD modelling studies, which usually address only the complex fluid dynamics of the process.

In this work, a three-dimensional CFD model for a stirred carbonation precipitation tank reactor was developed, aiming to investigate the process mechanistically, to identify the impact of the different mechanisms, and to study their interplay. The model is focused on the carbonation process within such reactors, which will serve as a basis for the incorporation of the chemical reactions leading to precipitation, which will be the subject of future work. In this context, the present model takes into account the flow dynamics of the liquid solution and the CO_2_ bubble flow. Furthermore, the mass transfer leading to the dissolution of CO_2,_ as well as its dissociation in water, are all taken into account. The model is validated with experimental measurements for the CO_2_ degassing rate and the pH within the solution, yielding a very good agreement. The analysis of the different phenomena and their interplay allows important mechanistic insight regarding the carbonation process. Finally, a parametric analysis is conducted, studying the effect of different process conditions on the mechanisms and the process dynamics. This analysis results in an in-depth understanding of the process and its underlying mechanisms, which in turn can potentially pave the way towards a knowledge-based process and reactor design, as well as assist the scale-up of carbonation and similar processes in stirred tank reactors.

## 2. Materials and Methods

### 2.1. Experimental Setup

**Figure 1:**
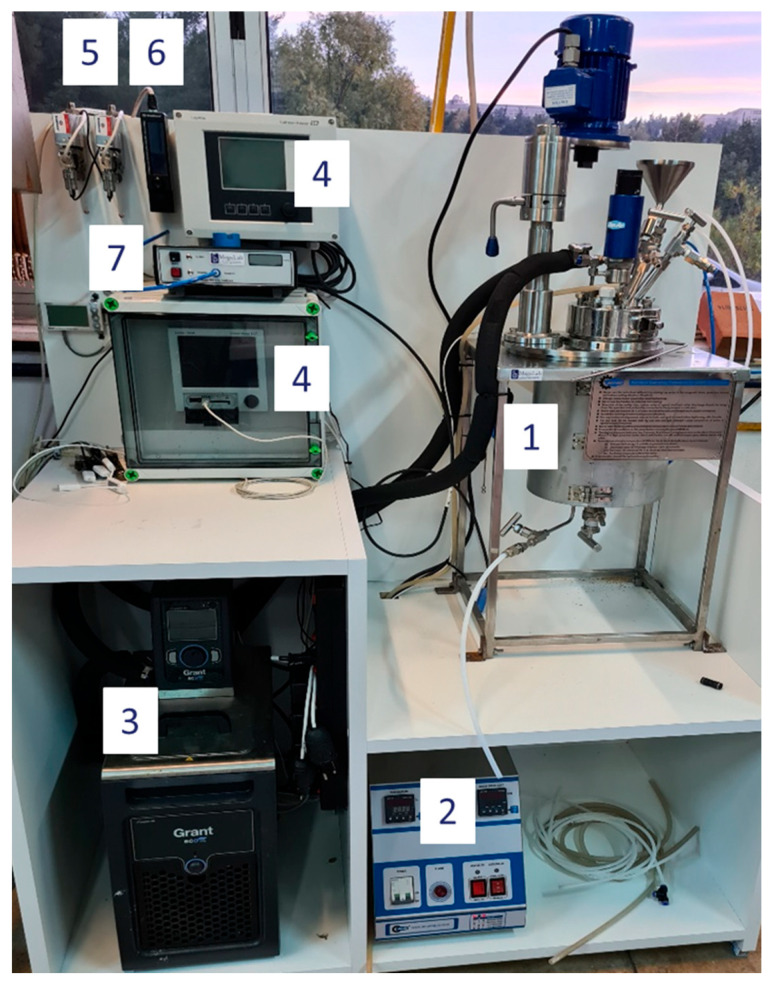
Experimental setup. (1) reactor, (2) controller, (3) chiller, (4) pH measuring system, (5) inflow mass flow controller, (6) outlet gas flowmeter, (7) CO_2_ analyzer.

The experiments were conducted with the experimental setup shown in Figure 1, which is a custom-made autoclave system by AMAR. The reactor (1) is made of Inconel 625 to resist highly alkaline solutions and has a 1.8 L working volume with a 102 mm diameter. It uses an electrical ceramic band for heating and a ¼ hp AC motor for stirring controlled by the controller (2). The vessel is equipped with baffles. The reactor has a flush bottom valve to unload the final pulp. Apart from the resistance heating, cooling or heating can also be controlled by a chiller (3) recirculating liquid through the cooling coil, which was removed for this model. The head of the reactor has a CO_2_ gas inlet, an outlet, a motorhead stirrer, an opening for sampling or for the pH electrode, a thermocouple inlet, and a funnel to insert solid or liquid samples. The stirrer type is a 6-bladed Rushton type with a 45° vane angle. The diameter of the stirrer is 50 mm. The impeller consists of two stirrers: one positioned at the bottom of the reactor and the other located approximately 1/3 of the way up from the bottom. This reactor is designed for the precipitation of solids through gas-liquid reactions and is intended to be compatible with pilot plant operations, which typically use stirred reactors. Therefore, a stirring reactor with a deep tube configuration was selected.

The pH is monitored and recorded by an Endress and Hauser measuring system (4). The gas flowrate is controlled with a 0–500 mLn/min mass flow controller by Bronkhorst, Monheim am Rhein, Germany, (F-501CV), calibrated for CO_2_ gas and controlled by computer software (5). The outlet is connected to a gas Bronkhorst flowmeter (MV-102) ranging from 0 to 2000 mL/min (6) and a CO_2_ analyzer (7) by Quantek instruments, Grafton, MA, USA, (model 908) ranging from 0 to 100% CO_2_ to measure the CO_2_ gas that is not consumed. Data for the CO_2_ outlet was taken every second.

The pH sensor is calibrated with certified buffers before each experiment using two appropriate buffers. Buffers with pH values of 4 and 9 were used. Each experiment was conducted at least twice to ensure reproducibility.

Pure carbon dioxide gas (99.995% purity) was used throughout all the experiments. The pH was monitored by a pH electrode by Endress and Hauser (Reinach, Switzerland), calibrated before each experiment, inserted into the solution equipped with a pH recorder, using a 1 min step.

A volume of 1100 mL of deionized water was introduced to the reactor, and the desired temperature was set. When the desired temperature is reached, firstly a stream of N_2_ gas is inserted into the reactor to ensure that no gas leakage takes place. Afterwards, CO_2_ gas was injected at a constant flow rate of 160 mL/min. The stirring rate was set at 200 rpm.

### 2.2. Computational Domain

**Figure 2:**
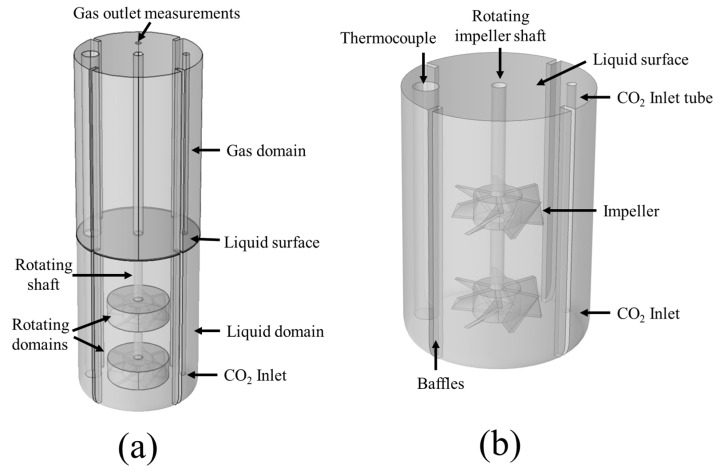
Computational domain and features for (**a**) the complete reactor model and (**b**) the liquid domain.

For the simulation of the carbonation process within the precipitation tank, two three-dimensional CFD models are developed. The first takes into account the phenomena occurring within the part of the tank that is filled with liquid (H_2_O, h = 142 mm), henceforth called the liquid domain model, while the second is developed for the space above the liquid surface filled with the gas, referred to as the gas domain model (see Figure 2). The reason for the incorporation of the gas domain model is to validate the developed computational framework with experimental measurements for the CO_2_ gas outflow, which are collected at the top of the precipitation reactor, at the CO_2_ outlet (Figure 2). The model geometry takes into account the various features of the precipitation tank, including the tank walls, the impeller shaft and blades of 50 mm diameter, the thermocouple, the CO_2_ inlet tube, and the tank baffles. Preliminary simulations showed minimal deformation for the gas-liquid interface due to the presence of the baffles, the thermocouple, and the inlet tube. This allowed the assumption of a stationary surface between the two domains. Finally, rotating domains around the region of the impeller blades are also defined to simulate the blade rotation, as in [38,39]. The complete computational domain for the precipitation reactor, as well as the liquid domain features, is shown below in Figure 2.

### 2.3. Governing Equations

As previously discussed, two different models are built for the liquid and gas domains. The liquid domain model simulates the multiphase flow within the precipitation tank, with the water flow due to the impeller rotation being coupled with the CO_2_ bubble flow within the water. Furthermore, the CO_2_ mass transfer between the gas and aquatic phases, the reactions that constitute the CO_2_ dissociation in water, as well as the dissolved species transport, are also taken into account by the liquid domain model. On the other hand, the gas phase domain takes into account the gas flow and mass transport of the different gas species. Both domains are assumed to be isothermal, as the experiments are conducted at constant temperatures. The governing equations that describe the different mechanisms and phenomena occurring within the different domains are presented in the following subsections.

#### 2.3.1. Liquid Domain Model

The analysis of the liquid domain begins with the assessment of the Reynolds number for the liquid flow in the precipitation tank, defined as:

(1) $$Re=\frac{ND^{2}}{\nu },$$

where N is the number of impeller rotations per time, D is the impeller blade diameter (50 mm), and ν is the kinematic viscosity. For the case of H_2_O flow within the tank, for a rotational speed of 200 rpm (as experimentally applied), the Reynolds number is deemed to be Re = 8325.

The multiphase flow is simulated in the present work assuming two distinct phases, liquid and gas (CO_2(g)_). As the gas flow is in the form of bubbles, it is simulated as a dispersed flow of uniform-sized spherical bubbles, while the gas-liquid interface (the interface between bubbles and liquid) is not simulated. This assumption significantly reduces the computational cost of the simulation, allowing faster simulations. As the present model is a phenomenological model, aiming to study the interplay between mass transfer and reaction mechanisms and their effect on the process macroscopically, this assumption is acceptable. Such approaches have been previously used to investigate the combined mass transfer and reactions in bubble flows in the environment [40,41,42,43], as well as in the case of chemical processes and reactors [17,32,44,45].

As the bubbles are assumed to be uniform in size, and the bubble dynamics such as breakage and coalescence are not the aim of the present study, the k-ε model is used for the modelling of the turbulent flow inside the stirring tank. The k-ε model turbulence model has been widely used for the CFD analysis of similar mixing tanks at comparable values of Re [31,39,46,47,48] due to its robustness and convergence and is therefore selected for the simulation of the H_2_O flow in the present work [31,49].

The multiphase flow is simulated using an Euler-Euler approach, with the two phases sharing the same pressure field. Finally, the model is developed under the reasonable assumption that the gas bubble density (~1 g/L) is negligible compared to the liquid density (~1000 g/L) and that the volume fraction of the gas phase is low in almost the entire liquid domain (except the region close to the CO_2_ inlet tube). The governing equations are presented below for an incompressible liquid, with the simplifications described in [43]:

Conservation of mass

(2) $$\rho_{l}\varphi_{l}\nabla \cdot u_{l}=0$$

Conservation of momentum

(3) $$\rho_{l}\frac{\partial }{\partial t}{\varphi_{l}u}_{l}+\rho_{l}\varphi_{l}u_{l}\cdot \nabla u_{l}=-\nabla P+\nabla \cdot \left[\varphi_{l}(\mu_{l}+\mu_{T})\left(\nabla u_{l}+\nabla u_{l}^{T}\right)\right]+\rho_{l}\varphi_{l}g$$

Gas phase transport

(4) $$\frac{\partial }{\partial t}\left(\rho_{g}\varphi_{g}\right)+\nabla \cdot \left(\rho_{g}\varphi_{g}u_{g}\right)=-m_{gl}$$

Conservation of dissolved species

(5) $$\frac{\partial C_{i}}{\partial t}-\nabla \;D_{i}\nabla \;C_{i}+u_{l}\nabla \;C_{i}=R_{i}$$

The turbulence is simulated with a standard k-ε model, using the following equations:

(6) $$\rho_{l}\varphi_{l}\frac{\partial k}{\partial t}+\rho_{l}\varphi_{l}u_{l}\cdot \nabla k=\nabla \cdot \left[\varphi_{l}\left(\mu_{l}+\frac{\mu_{T}}{\sigma_{\kappa }}\right)\nabla k\right]+p_{k}-\varphi_{l}\rho_{l}\varepsilon$$

(7) $$\rho_{l}\varphi_{l}\frac{\partial \varepsilon }{\partial t}+\rho_{l}{\varphi_{l}u}_{l}\cdot \nabla \varepsilon =\nabla \cdot \left[\varphi_{l}\left(\mu_{l}+\frac{\mu_{T}}{\sigma_{\varepsilon }}\right)\nabla \varepsilon \right]+{C_{\varepsilon 1}\frac{\varepsilon }{k}p}_{k}-C_{\varepsilon 2}\frac{\varepsilon^{2}}{k}{\varphi_{l}\rho }_{l}$$

Finally, the volume fraction constraint is imposed:

(8) $$\varphi_{g}+\varphi_{l}=1$$

In Equations (2)–(8), subscripts l and g refer to the liquid and gas phases, respectively. ρ is the density, **u** is the velocity vector, P is the pressure, and φ is the volume fraction of the corresponding phase. The variables k and ε are the kinetic energy and dissipation rate, respectively, while μ_l_ is the laminar viscosity and m_gl_ is the mass transfer rate between the two phases. p_k_ expresses the turbulence production due to buoyancy and viscous forces, while μ_T_ is the turbulence viscosity. Both p_k_ and μ_T_ are computed as in [31], using the same turbulence parameter values for C_μ_, C_ε1_, C_ε2_, σ_κ_, and σ_ε_. Finally, C_i_ is the concentration of dissolved species i, D_i_ is the diffusion coefficient for dissolved species in H_2_O, and R_i_ is the production/consumption rate of each dissolved species due to mass transfer and/or chemical reactions.

Following the above formulation, the approach for the bubble flow simulation lies in the definition of the gas bubble velocity, u_g_. This is done by analyzing the different forces that are imposed upon the CO_2_ bubbles during their flow through the liquid, as in [50]. However, for the dispersed flow of bubbles with a small diameter, the dominant forces are usually deemed to be those exerted by the liquid pressure and the drag forces due to the bubble movement, reducing the approach to a pressure-drag balance [41,42,50,51,52]. Therefore, the definition is restricted to the following two equations:

(9) $$u_{g}=u_{l}+u_{slip}$$

(10) $$\varphi_{l}\nabla \;p=-\varphi_{l}C_{D}\frac{3\rho_{l}}{4d_{b}}\left|u_{slip}\right|u_{slip},$$

where u_slip_ is the relative velocity between the bubble and the liquid, and d_b_ is the mean bubble diameter. In the present case, the drag coefficient is computed based on the approximation of Schwartz and Turner for bubble flow in stirred tanks for bubble diameters of 1–10 mm [49], which has been successfully used in previous works [17,53].

Besides the CO_2_ bubble flow, the CO_2_ mass transfer between gas and liquid phases is also simulated by introducing the source term of m_gl_ in Equation (4). The mass transfer between the two phases is computed using the two-film theory approach for the CO_2_ solubility in water:

CO_2(g)_ ↔ CO_2(aq)_ (R1)

(11) $$m_{gl}=k_{m}\cdot \left(C_{CO2,sol}-C_{CO2,l}\right)\cdot a,$$

where k_m_ is the mass transfer coefficient, C_CO2,l_ is the concentration of the dissolved CO_2(aq)_ in the liquid, and C_CO2,sol_ is the solubility of CO_2_ in H_2_O, estimated by Henry’s law:

C_CO2,sol_ = P_CO2_H_CO2_, (12)

where P_CO2_ is the partial pressure of CO_2(g)_, and H_CO2_ is Henry’s constant for the CO_2_-H_2_O system.

In Equation (11), α denotes the total surface area per unit volume of the CO_2_ bubbles, computed as α = A_b_∙N_b_, where A_b_ is the surface area of a single bubble and N_b_ is the number density of bubbles:

A_b_ = πd_b_^2^ (13)

(14) $$N_{b}=\frac{\varphi_{g}}{V_{b}},where\;V_{b}=\frac{4\pi r_{b}^{3}}{3}$$

The mass transfer coefficient is computed based on the approach presented in previous works [40,42,54].

(15) $$k_{m}=\frac{2}{\sqrt{\pi }}\sqrt{Sc{Re}_{b}}\frac{D_{co2}}{d_{b}},$$

where D_CO2_ is the diffusion coefficient of CO_2_ in water, Sc is the turbulent Schmidt number, and Re_b_ is the Reynolds number of the bubble.

(16) $${Re}_{b}=\frac{\rho_{l}\left|u_{slip}\right|d_{b}}{\mu_{l}}$$

Once the CO_2(aq)_ is dissolved into the liquid, CO_2_ hydrolysis takes place. As H_2_O is in abundance within the liquid, the hydrolysis is expressed without the use of reaction intermediaries, under the following mechanisms:

CO_2(aq)_ ↔ H^+^ + HCO_3_^−^ (R2)

HCO_3_^−^ ↔ H^+^ + CO_3_^−2^ (R3)

For the first dissociation reaction, first-order kinetics are imposed for the backward and forward rates, from which the equilibrium constant can be computed [55]:

(17) $$R_{f}=k_{f}C_{CO2(aq)}$$

(18) $$R_{b}=k_{b}C_{H}C_{HCO3}$$

(19) $$K=\frac{k_{f}}{k_{b}}$$

The second dissociation reaction (R3) is known to be significant only in higher pH, since the equilibrium constant is very low, often reported in the range of 10^−9^–10^−11^ [21,55]. For this reason, it is not taken into account by the present model.

#### 2.3.2. Gas Domain Model

The phenomena taking place within the gas phase domain include gas flow and species transport. The gas flow within the gas domain is characterized by lower density and lower gas velocities, resulting in a lower Reynolds number (Re < 100). For this reason, the gas flow is simulated as a Newtonian fluid assuming laminar flow, as in [56,57,58,59,60].

Conservation of mass

(20) $$\frac{\partial \rho_{g}}{\partial t}=\nabla \cdot \left(\rho_{g}u_{g}\right)=0$$

Conservation of momentum

(21) $$\frac{\partial (\rho_{g}u_{g})}{\partial t}+\nabla \cdot \left(\rho_{g}u_{g}u_{g}\right)=-\nabla P+\nabla \cdot \left[\mu_{g}\left(\nabla u_{g}+\nabla {u_{g}}^{T}\right)-\mu_{g}\frac{2}{3}\left(\nabla \cdot u_{g}\right)I\right]+\rho_{g}g$$

The conservation of chemical species in the gas phase is modelled using the following equation [61]:

Conservation of chemical species

(22) $$\frac{\partial (\rho \omega_{i})}{\partial t}+\nabla \cdot \left(\rho u\omega_{i}\right)=-\nabla \cdot j_{i}$$

where ω_i_ is the mass fraction of the i species in the gas phase, ρ is the gas mixture density, while the diffusion flux **j_i_** is calculated:

(23) $$j_{i}=-\rho \omega_{i}\sum_{k=1}^{n-1}D_{ik}\left[\nabla x_{k}+(x_{k}-\omega_{k})\frac{\nabla P}{P}\right],$$

where D_ik_ is the binary diffusion coefficient and x_k_ is the mole fraction.

The gas properties, such as the gas mixture viscosity and the binary diffusion coefficient, are computed using the kinetic gas theory [62,63], using the Lennard-Jones parameters for the species from the CHEMKIN-PRO database [64]. Finally, the gas mixture density is calculated using the ideal gas law.

### 2.4. Model Implementation

#### 2.4.1. Boundary Conditions

For the simulation of the experimental process with the corresponding operating conditions, the appropriate boundary conditions must be implemented on the governing equations presented in the previous sections. For the liquid domain, the tank walls and baffles, as well as the thermocouple and CO_2_ inlet tube walls, are simulated using a no-slip condition for the liquid flow, while a zero flux is imposed for the gas phase and dissolved species conservation equations. A CO_2(g)_ inlet flow equal to the experimental (160 sccm) is imposed on the CO_2_ inlet tube. A rotational speed (200 rpm according to the experiments) is imposed on the impeller shaft walls and the rotating domains around the impeller blades, as shown in Figure 2. Regarding the liquid surface simulation, preliminary computations were conducted using a free surface boundary condition by imposing the appropriate surface tension coefficient and an ambient pressure in order to examine whether surface deformation through vortex formation occurs. The preliminary simulations showed no vortex formation and minimal surface deformation due to the presence of the baffles, the thermocouple, and the inlet tube. Based on these preliminary results on the liquid surface, a different approach was used for subsequent simulations, using a slip wall condition for the liquid flow. In this way, the computationally expensive calculation of the surface deformation is not taken into account, while the rotational velocity of the liquid on its surface is preserved for the simulation of the liquid surface. For the dissolved species, a zero-flux condition is imposed on the surface, while for the simulation of the CO_2(g)_ degassing, it is assumed that the bubbles on the surface with a vertical relative velocity towards the gas domain are degassed. This is done by imposing the following flux on the liquid surface:

(24) $$J_{b}=u_{slip,z}\rho_{g}\varphi_{g}$$

For the gas phase domain, a no-slip, no-flux wall condition is imposed on the domain walls. An outlet atmospheric pressure and zero flux are imposed on the CO_2(g)_ outlet, while the experimental rotational speed is imposed on the impeller shaft walls. The models for the gas and liquid domains, respectively, exchange information using a one-way coupling approach, under the assumption that the CO_2(g)_ that is degassing from the liquid surface cannot redissolve back to the H_2_O liquid solution. Under this assumption, the flux computed by Equation (24) is imposed as a CO_2(g)_ inlet condition on the liquid-gas interface (liquid surface).

#### 2.4.2. Simulation Procedure

**Figure A1:**
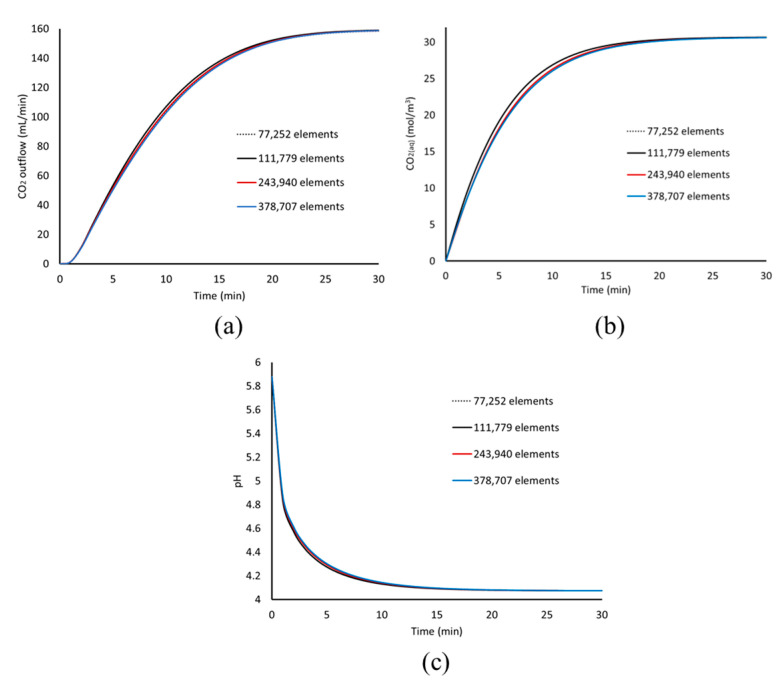
Model results for the different computational meshes used for the simulation. (**a**) CO_2_ outflow, (**b**) CO_2(aq)_ concentration, and (**c**) pH as a function of time.

**Figure A2:**
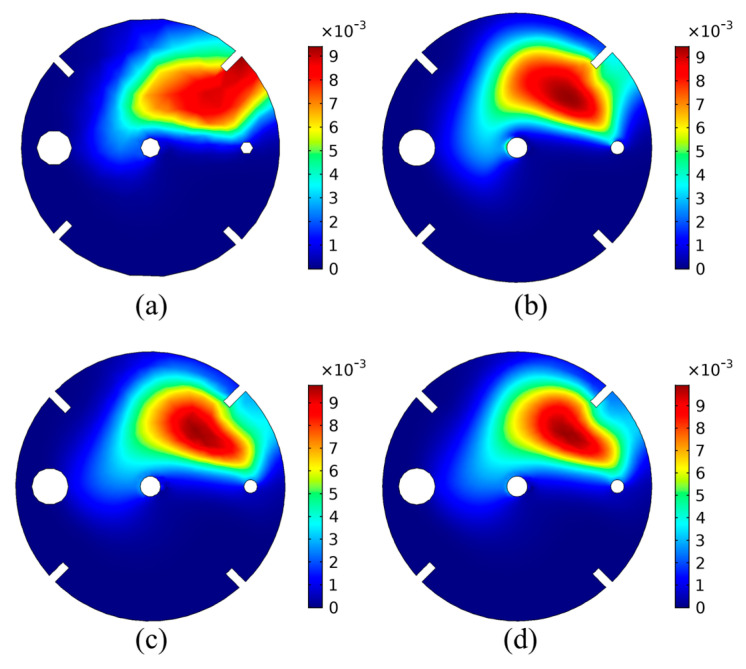
Results of the CO_2(g)_ bubble volume fraction on the liquid surface, for different mesh sizes: (**a**) 77,252 elements, (**b**) 111,779 elements, (**c**) 243,940 elements, (**d**) 378,707 elements.

**Figure A3:**
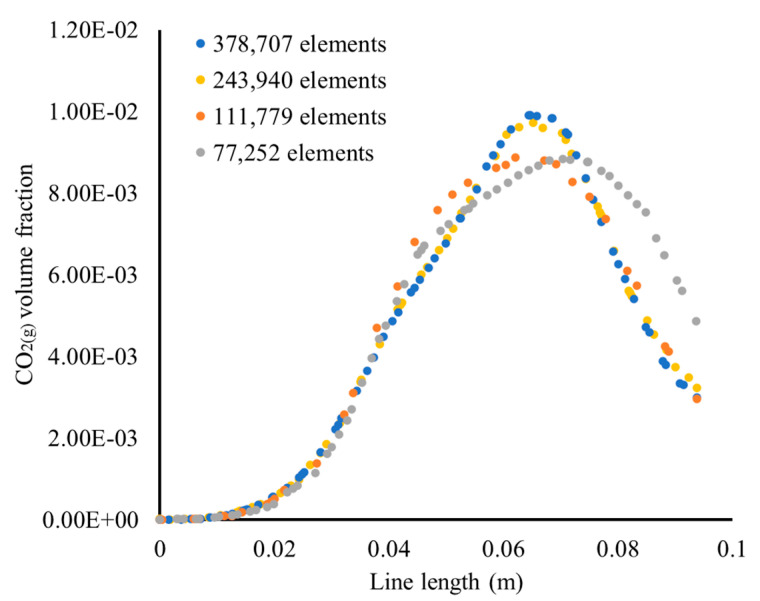
Results of the CO_2(g)_ bubble volume fraction on the liquid surface, along a horizontal line passing through the region of the maximum volume fraction.

**Figure A4:**
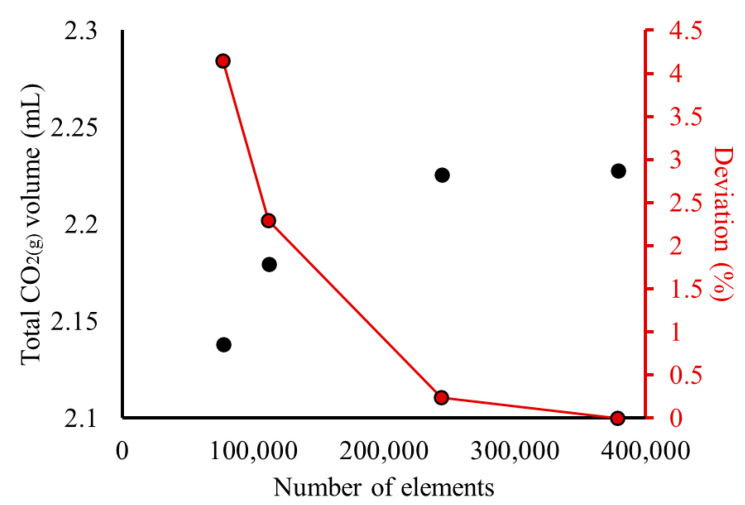
Total volume of the CO_2(g)_ bubbles in the stirring tank (black circles, black axis) and deviation percentage (red circles, red axis), as a function of the mesh size.

The governing equations presented in the previous sections are discretized and solved with the finite element method, using Comsol Multiphysics 5.5^®^, over a computational mesh of 243,940 elements. The computational mesh was selected following a grid independence study, shown in the Appendix A, in Figure A1, Figure A2, Figure A3 and Figure A4. The initial solution for the liquid domain assumes zero velocity of H_2_O, without any CO_2(g)_ inlet flow, with an initial concentration of H^+^ set according to the pH measurements. The gas domain is assumed to be filled with atmospheric air of zero velocity, under atmospheric pressure. The properties of atmospheric air are computed based on the kinetic gas theory using the Lennard-Jones parameters for N_2_.

The liquid domain is first simulated without the CO_2(g)_ inlet flow, using a frozen rotor or multiple reference frame computation, which allows the estimation of a pseudo-steady state solution for the rotational flow within the liquid domain. Then, the CO_2(g)_ inlet flow is imposed, and the process is simulated using a time-dependent study in order to simulate the whole range of phenomena.

Finally, although the CO_2_ inlet tube has a diameter of 5 mm, the actual mean size of the bubbles in the liquid is smaller due to the agitation of the liquid and the CO_2_ dissolution. Therefore, the mean bubble diameter (d_b_) is used as a fitting parameter, with a maximum bubble size of 5 mm, equal to the CO_2_ inlet tube diameter.

The parameter values used for the simulation of the different phenomena and mechanisms presented in the previous sections are summarized in Table 1.

## 3. Results and Discussion

### 3.1. CFD Analysis of the Precipitation Tank

**Figure 3:**
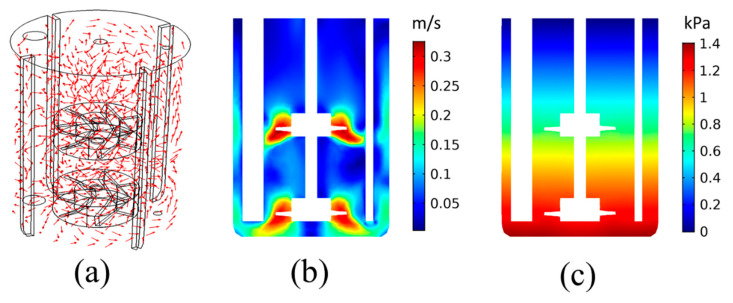
Results for the pure H_2_O flow: (**a**) Flow field within the precipitation tank, (**b**) velocity magnitude, and (**c**) relative pressure distribution on a cross-sectional plane.

The first step of the analysis considers the investigation of the fluid dynamics within the liquid part of the precipitation reactor. For this reason, the rotating flow of liquid H_2_O, without any CO_(g)_ inflow, is simulated at T = 30 °C. The resulting flow field and relative pressure distribution are presented in Figure 3. It is noted that the relative pressure is shown with a reference pressure of 1 atm.

Figure 3a shows that the flow field is mainly dominated by the rotating impeller dynamics, as expected. The velocity vector follows the direction of the impeller rotation on most parts of the tank. The velocity is higher closer to the impeller blades, as shown in Figure 3b. It is noted that the small blade height shown in Figure 3b is due to the vertical cross-sectional plane used for the results representation and the fact that the impeller blades are inclined. The lower velocities are computed closer to the liquid surface, as well as between the tank bottom and the lower part of the impeller shaft. The pressure distribution is dominated by the hydrostatic pressure, with pressure linearly increasing with the depth, as shown in Figure 3c. This shows that the main pressure gradient is along the tank height. According to Section 2.3, the pressure-drag balance dominates the bubble dynamics (Equation (10)). As Figure 3c shows that gravitational forces dominate the pressure field, this would mean that the main driving force for the CO_2(g)_ bubbles would be the buoyancy forces.

**Figure 4:**
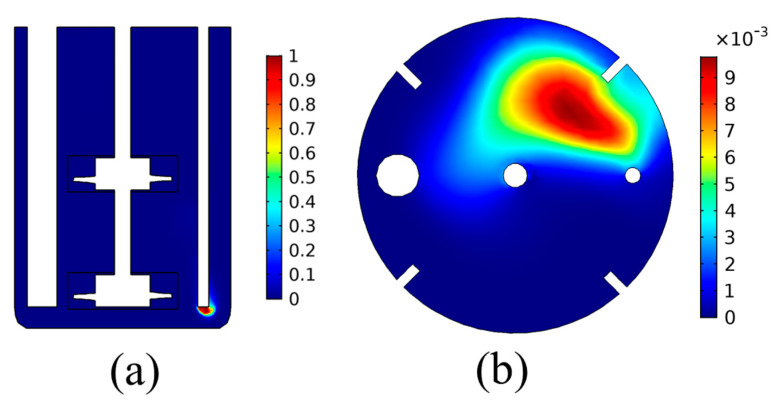
CO_2(g)_ volume fraction distribution: (**a**) cross-sectional plane, (**b**) liquid surface.

For the study of the bubble dynamics, the analysis proceeds with the simulation of the precipitation tank dynamics, including the CO_2(g)_ flow, for the same temperature of T = 30 °C. The results for the gas bubble volume fraction, obtained by the pseudo steady state simulation using the frozen rotor approach, are presented in Figure 4.

The results of Figure 4 show that the volume fraction of the gas bubbles is low in almost the entire domain of the precipitation tank, except for the zone close to the CO_2(g)_ inlet. This shows that the bubbles are quickly dispersed into the liquid once they are injected into the tank. Furthermore, it is seen that the bubble movement is mainly drifted by the hydrostatic pressure distribution. As can be seen by the results of Figure 4b, the bubble volume fraction on the liquid surface is higher close to the inlet tube, showing that the vertical velocity of the bubbles is higher than the other velocity components due to buoyancy. This hints that the buoyancy forces due to the density difference between the gas bubble and the liquid dominate the convection imposed by the liquid’s rotational motion. Such results can provide significant insight regarding the design of carbonation precipitation tanks regarding the position of the CO_2(g)_ inlet tube. For example, other works have presented the design of carbonation reactors with gas inlets on the bottom of the tank or the impellers, as well as on the impeller blades, to achieve better CO_2_ bubble dispersion [31].

The results so far illustrate the significance of such computational approaches to provide insights into the different phenomena occurring during the process. The results presented in this section consider the pseudo-steady state of the process, meaning that the flows are stabilized, the liquid solution is saturated with CO_2(aq)_, and the CO_2_ hydrolysis reactions have reached equilibrium. The effect of the fluid dynamics, however, affects the kinetics of the abovementioned processes, i.e., a dynamic analysis of the process is required to obtain mechanistic information regarding CO_2_ dissolution and hydrolysis within the H_2_O solution. This analysis is presented in the next section.

### 3.2. CO_2_ Bubble Dynamics, Dissolution, and Hydrolysis

**Figure 5:**
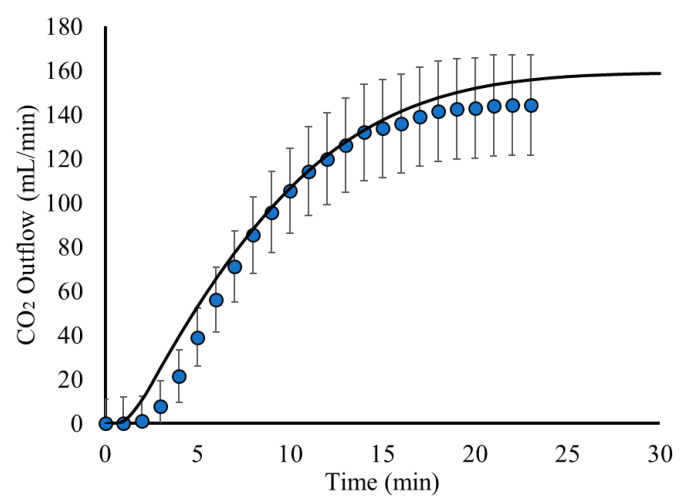
CO_2(g)_ outflow as a function of time: Model predictions (black line) compared to experimental measurements (blue circles).

For the investigation of the CO_2(g)_ bubble dynamics, the process is studied using a time-dependent simulation, using the results for the pure H_2_O flow as an initial condition (Figure 3). The gas phase domain is also simulated to compare with experimental measurements for the CO_2(g)_ outflow from the reactor. The results regarding the CO_2(g)_ outflow are presented in Figure 5.

Figure 5 shows that the model results for the CO_2(g)_ outflow are in good agreement with the experimental measurements. This shows that the model is able to capture the bubble flow dynamics with good accuracy. As it is shown, the CO_2(g)_ outflow is minimal during the first minutes. This is explained by the fact that the bubbles need time to disperse and flow within the liquid, as well as degas from the liquid surface. Furthermore, the CO_2(g)_ dissolution rate is higher during the first minutes since there is less dissolved CO_2(aq)_ in the liquid solution. Thus, the mass transfer from the gas to the aquatic phase is higher during the first minutes, leading to less CO_2(g)_ exiting the reactor. After a few minutes, however, the dissolution of CO_2(g)_ decreases, leading to higher amounts of CO_2_ bubbles degassing and leaving the surface of the liquid and the reactor. This can be seen in Figure 5, where an abrupt increase in the CO_2(g)_ outflow after the first minutes is observed. During this time, the gas domain of the reactor is slowly filled with CO_2(g)_, while the atmospheric air flows out of the reactor. This continues until the pseudo steady state of the process is attained, where the liquid solution is saturated with CO_2_ and all the CO_2_ bubbles degas and leave the reactor at a constant rate, as shown in the results of Figure 5.

**Figure 6:**
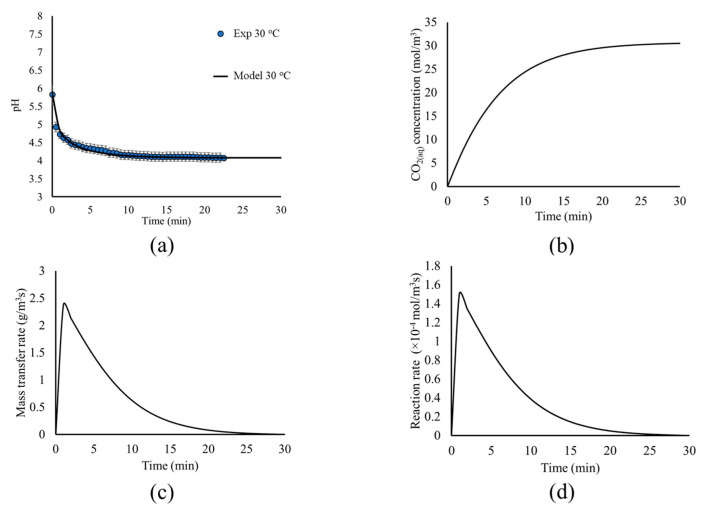
(**a**) Model predictions (black line) and experimental measurements (blue circles) for the pH evolution, (**b**) CO_2(aq)_ concentration, (**c**) mass transfer rate, and (**d**) reaction rate, as a function of time.

The kinetics of the above-mentioned physical and chemical mechanisms are illustrated in Figure 6a,b, where the pH and CO_2(aq)_ concentration are presented as a function of time, respectively. The model predictions are plotted in comparison with the experimental measurements for the pH within the tank. The results for the rates of mass transfer and chemical reaction (R2) are also plotted in Figure 6c,d, respectively.

Figure 6a shows that the model predictions for the pH evolution within the tank are in excellent agreement with the experimental measurements; thus, validating the approach for the simulation of the CO_2(g)_ bubble flow, dissolution, and hydrolysis and the choice of the corresponding kinetic expressions presented in Table 1. The pH evolution shows a very abrupt decrease of pH during the first minutes, explained by the rapid dissolution and hydrolysis of CO_2(g)_, due to the fact that the system is far from equilibrium. As minimal CO_2_ is initially dissolved in the water, the mass transfer rate from the gas phase to CO_2(aq)_ is higher during the first minutes, as seen in Figure 6c. In turn, CO_2(aq)_ dissociates to HCO_3_^−^ and H^+^, as the equilibrium favors the dissociation (Figure 6d), lowering the pH. As the concentration of HCO_3_^−^ and H^+^ increases, the system approaches equilibrium, and the dissolution rate decreases (Figure 6d), leading to the pH decreasing more slowly. This allows higher CO_2(aq)_ concentrations within the liquid solution (Figure 6b), which in turn decreases the mass transfer rate from the bubbles to the liquid solution, as seen in Figure 6c. This process continues until the system reaches chemical and physicochemical equilibrium, where the concentrations of the dissolved species are dictated by the equilibrium constant and Henry’s law. This also allows the process to reach its steady state in terms of fluid dynamics, where the mass transfer rate between the two phases is minimal.

The results of this section, presented in Figure 5 and Figure 6, show the effect of the interplay between the different mechanisms on the process dynamics. In particular, the liquid flow within the mixing tank due to the impellers, the CO_2(g)_ bubble dynamics, the mass transfer rate between the two phases, and the CO_2(aq)_ dissociation all influence the dynamics of the whole process. It is this interplay that dictates the carbonation behavior under different process conditions and should be taken into account for process design and optimization and the interpretation of experimental measurements. The in-depth understanding and mechanistic insight are challenging to obtain using only experimental measurements and observations, as the abovementioned phenomena are coupled and simultaneous, while they also take place in different time scales. However, the development of computational models, such as in the present case, allows this mechanistic overview of the different mechanisms and their interplay and can be used to draw conclusions regarding the dominating mechanisms of the carbonation process.

### 3.3. Effect of Process Conditions

Based on the above analysis and understanding of the underlying carbonation mechanisms, the investigation proceeds with the study of the effect of different process parameters. The process parameters under study are the temperature, CO_2_ inlet flow rate, and the impeller rotational speed. The effect of each process parameter is presented in the following subsections.

#### 3.3.1. Effect of Temperature

**Figure 7:**
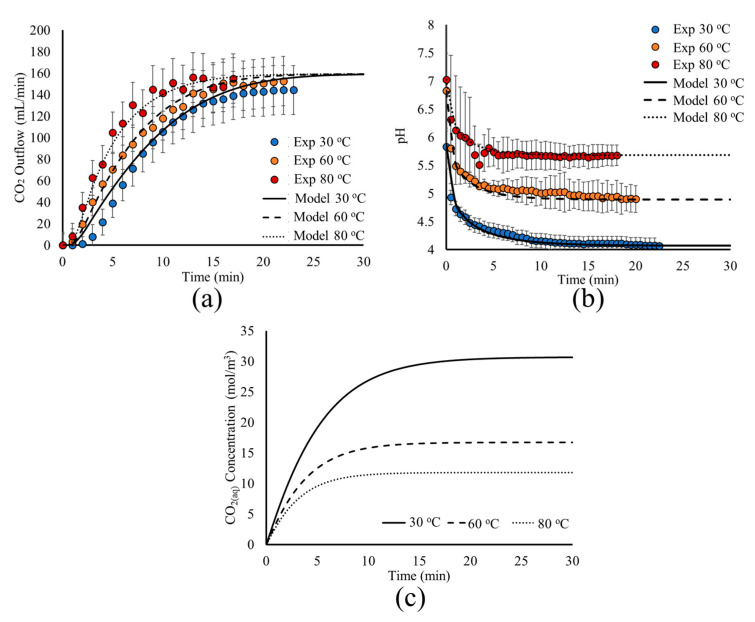
Model predictions (lines) and experimental measurements (circles) for the effect of temperature on the time evolution of (**a**) CO_2(g)_ outflow, (**b**) pH, and (**c**) CO_2(aq)_ concentration within the tank.

To study the effect of temperature on the carbonation process, the model is simulated under a time-dependent study for the temperatures of T = 30 °C, 60 °C, and 80 °C. The results of the analysis are presented in Figure 7a,b, where the model predictions for the CO_2(g)_ outflow and pH are presented and compared with experimental measurements as a function of time. The CO_2(aq)_ concentration as a function of time is also presented in Figure 7c.

Figure 7 shows that the model predictions are in very good agreement with experimental measurements for both the CO_2(g)_ outflow (Figure 7a) and pH (Figure 7b) evolution as a function of time for the three different temperature values. For higher temperatures, the time required for the system to reach its pseudo steady state is decreased. This is shown by the time needed to reach the constant CO_2(g)_ outflow rate and pH.

This behavior is explained by the analysis of the underlying mechanisms. First, the temperature impacts the fluid dynamics and the CO_2(aq)_ transport, affecting the liquid viscosity and CO_2(aq)_ diffusion (Table 1). Furthermore, temperature increase favors the fast transport of the CO_2(g)_ that is degassed from the liquid surface towards the reactor outlet, since it impacts the diffusion coefficient and viscosity of the gas mixture, as shown in Table 1, and by the results of Figure 7a.

However, the main impact of temperature is on the dissolution and dissociation mechanisms. At higher temperatures, the mass transfer constant from the gas bubbles to the liquid phase increases. Furthermore, a higher temperature leads to a lower Henry constant for CO_2_, that is, the liquid solution is saturated at a lower CO_2(aq)_ concentration, decreasing the maximum concentration of CO_2(aq)_ that can be achieved. This behavior can be seen in Figure 7c, where the liquid solution saturation to CO_2(aq)_ is achieved faster and at a lower concentration when the temperature is increased. Finally, although the rates for the dissolution reactions are favored by the temperature increase, the reaction equilibrium constant is lower, shifting towards the CO_2(aq)_ side. This is due to the higher activation energy of the backward rate in Equation (18), which leads to a higher temperature dependence (Table 1). Hence, both the physicochemical and chemical equilibria are achieved faster.

The effect of temperature on the above mechanisms is illustrated in Figure 7a, where a more abrupt increase of the steady CO_2(g)_ outflow is obtained at higher temperatures. Furthermore, in Figure 7b, a rather gradual decrease in pH is observed, explained by the lower dissolution rate due to the smaller concentration difference between the saturation concentration and the liquid solution (Figure 7c). More importantly, higher temperatures result in higher pH values due to the dissociation reaction equilibrium being less favorable towards H^+^ production (Equation (R2)).

The presented analysis showed that temperature has an overall contribution to the process dynamics, with its main effect being on the physicochemical and chemical equilibrium, affecting their interplay with chemical kinetics and bubble flow dynamics. These results illustrate the capability of such computational approaches to identify dominating mechanisms within complex processes such as the carbonation precipitation, assisting process optimization through the mechanistic understanding of process parameter effects, such as temperature.

Regarding the model validation, although local measurements are not obtained regarding the local velocities, gas holdup distribution, and dissolved CO_2_ concentration, the model is validated with two separate measurements. The pH evolution is indicative of the dissolution and reaction kinetics within the reactor, while the CO_2_ outflow measurements present an indicative result for gas holdup and bubble flow. Therefore, the model is validated, although not locally, for both the dissolution-reaction kinetics and the bubble transport for different temperature values. Taking into account that the present approach is a phenomenological one, the validation of the model using these two separate measurements as a function of time, for three different temperatures, makes the approach reliable.

#### 3.3.2. Effect of CO_2_ Inflow Rate

**Figure 8:**
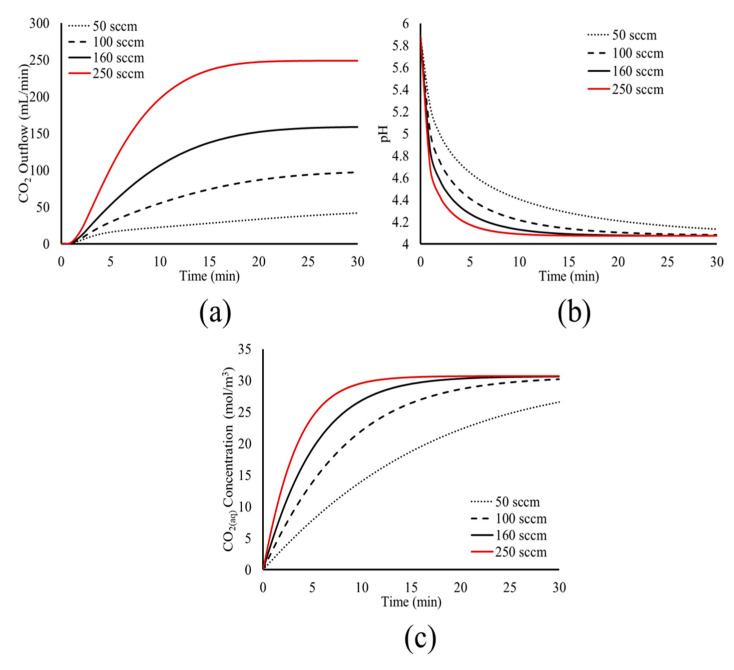
Model predictions for the effect of CO_2_ inflow rate on the time evolution of (**a**) CO_2_ outflow, (**b**) pH, and (**c**) CO_2(aq)_ concentration within the tank.

Following the study of the temperature effect, the analysis proceeds with the investigation of the CO_2_ inlet flow. The model predictions for the CO_2_ outflow, the pH, and the CO_2(aq)_ concentration for a varying CO_2_ inflow rate and at constant T = 30 °C, are presented in Figure 8.

Figure 8 shows that the CO_2_ inflow rate affects the dynamics of the carbonation process; a higher CO_2_ inflow rate leads to less time required for the system to reach its pseudo-steady state. The higher inflow rate increases the volume fraction of bubbles within the liquid, increasing the dissolution rate, since more bubbles interact with H_2_O via their surface. This is shown in Figure 8c, where the CO_2(aq)_ concentration increases more rapidly at higher CO_2_ inflow rates. In turn, the faster dissolution of CO_2_ leads to faster dissociation, shown by the more abrupt decrease of the pH value in the tank, as seen in Figure 8b.

The faster dissolution and dissociation of CO_2(aq)_ at higher CO_2_ inflow rates leads to less time needed to reach the physicochemical and chemical equilibrium. Following the results of Figure 8, for the CO_2_ inflow rates of 100 sccm, 160 sccm, and 250 sccm, the time needed for equilibrium is 30 min, 20 min, and 10 min, respectively. For the case of 50 sccm, the simulation time of 30 min was not enough to reach equilibrium.

Once the equilibrium is reached within the tank, the bubble flow within the liquid and the CO_2_ degassing stabilize, and the system reaches its pseudo steady state, where the dissolution of CO_2(g)_ bubbles is minimal and the flow field is stable. This is shown in Figure 8a, where higher CO_2_ inflow rates lead to less time needed to reach the pseudo steady state of the system, with a constant CO_2_ outflow.

These results show the effect of the fluid dynamics and transport phenomena on the carbonation process and its kinetics. Such results should be taken into account for process design and optimization, as they can provide mechanistic insight and information and assist in the control and fine-tuning of the different aspects of the carbonation process, according to the specific applications.

#### 3.3.3. Effect of Impeller Rotational Speed

**Figure 9:**
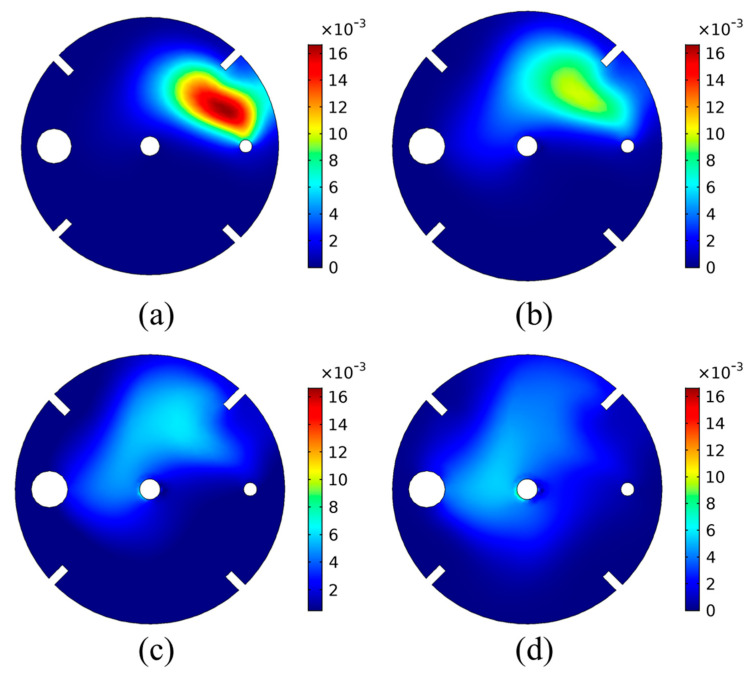
Volume fraction of CO_2(g)_ bubbles on the liquid surface for varying impeller rotational speed: (**a**) 150 rpm, (**b**) 200 rpm, (**c**) 250 rpm, (**d**) 300 rpm.

Besides the effect of temperature and the CO_2_ inflow rate, the effect of the impeller rotational speed was also investigated. The reactor was simulated using rotational speeds of 150, 200, 250, and 300 rpm at a constant T = 30 °C. It should be noted that the effect of the rotational speed on the bubble size could not be investigated using the present approach, as the bubble size is considered uniform. Under the uniform bubble size assumption, the effect of the rotational speed on the CO_2_ outflow, the overall pH evolution, and the overall CO_2_ concentration in the liquid solution was minimal and therefore not shown. Therefore, detailed results regarding the bubble dispersion cannot be investigated using the current approach, which is a drawback of the model. Nevertheless, a significant effect was found on the bubble and species distribution within the tank, even when assuming a constant bubble size. This is shown in Figure 9, where the CO_2(g)_ bubble volume fraction on the liquid surface is plotted for the varying rotational speed.

As shown in Figure 9, for lower impeller rotational speed, the liquid’s rotational motion is slower, leading to lower drag forces being imposed on the bubbles by the liquid. This leads to less bubble dispersion within the tank. On the contrary, higher rotational speed leads to higher liquid velocity, which in turn yields higher bubble dispersion.

For the case of 150 rpm (Figure 9a), it is seen that the CO_2(g)_ bubbles are situated in a zone closer to the CO_2_ inlet tube, under higher volume fractions. This means that the flow of bubbles to the surface due to buoyancy is the most significant contributor to the bubble flow. The liquid velocity due to the impeller rotation has a smaller effect on the bubble flow, and thus the bubble dispersion in the tank is limited. Impeller rotational speed increase leads to a different volume fraction profile on the liquid surface for the CO_2(g)_ bubbles. As seen in Figure 9b–d, the increase in the rotational speed increases the bubble dispersion within the tank. A lower volume fraction of CO_2(g)_ bubbles is obtained but over a larger area of the liquid surface. This is due to the higher liquid velocity caused by the increased rotational speed of the impellers, which increases the forces inflicted on the bubbles. It is the balance between these forces and the buoyancy that dictates the bubble flow and hence their dispersion.

**Figure 10:**
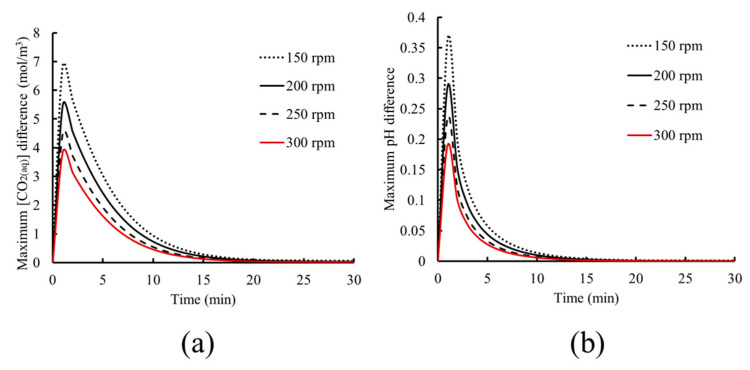
Evolution of the maximum difference within the tank for (**a**) CO_2(aq)_ concentration and (**b**) pH.

These results show that although the average kinetics and dynamics within the tank may not be affected by the rotational speed, the local concentrations of chemical species and the bubble volume fraction are influenced, and consequently, the dissolution and dissociation rates may vary. To illustrate this behavior, the maximum difference between the CO_2(aq)_ concentration and pH within the tank, defined as the difference between the maximum and minimum values within the liquid domain, is presented in Figure 10 as a function of time.

As the results of Figure 10 indicate, lower rotational speed leads to higher variations of CO_2(aq)_ concentration and pH within the tank during the time duration required for the system to reach saturation. This is explained by the lower bubble dispersion at low rotational speed, leading to a higher bubble volume fraction. In turn, this results in local dissolution and subsequent dissociation of CO_2(aq)_, causing a higher local concentration and pH variation. As time progresses, this variation gradually decreases since the dissolved chemical species diffuse in the liquid, yielding a more uniform distribution of chemical species concentration. This continues until the liquid solution is saturated and chemical equilibrium is achieved, resulting in uniform species distribution within the tank and allowing the system to reach its pseudo steady state.

An increase in the rotational speed leads to lower concentration variations, as seen in Figure 10. This is due to the higher bubble dispersion due to the liquid’s rotational motion being increased, as previously seen in Figure 9. This leads to dissolution and dissociation taking place in wider regions within the fluid because of bubble dispersion within the liquid. Furthermore, the transport of chemical species is enhanced through the higher convection due to the higher liquid momentum for higher rotational speed. All the above phenomena lead to a shorter time needed to achieve physicochemical and chemical equilibrium, resulting in uniform species distribution.

Although the abovementioned effects are not shown when the overall pH or CO_2(aq)_ concentration within the liquid solution is monitored, the local variations of bubble volume fraction and chemical species dissociation can play an important role in local reaction rates. These mechanisms affect specific phenomena, such as precipitation, with the local formation and coalescence of reaction (by)products, yielding solid particle formation precipitating to the tank bottom. Furthermore, local pH increase due to dissociation may activate certain secondary reaction mechanisms. These phenomena can be desirable or not, depending on the chemical process in question. Finally, rotational speed may also have an influence on bubble size, which for the present model is assumed to be uniform across the domain, and its variation is not taken into account by the model. The effect of the abovementioned observation on phenomena such as precipitation requires the inclusion of other chemical species and reactions within the model development to simulate such kinds of processes, which is not the scope of the present work.

It is clear that this level of insight regarding the local concentration variations within the tank is very challenging to obtain experimentally, which demonstrates the capacity and necessity of such computational approaches to unravel the effect of phenomena similar to those presented. The identification and understanding of the underlying mechanisms of local concentration variations can assist the optimization of the process to increase or reduce their effect, according to the required application.

#### 3.3.4. Influence of Process Parameters

The results of this section showed the effect of three key process parameters, namely the temperature, CO_2_ inflow rate, and rotational speed. Results showed that temperature had a significant influence on the carbonation process via its effect on the equilibrium for both the CO_2_ dissolution and dissociation. Temperature increase led to the decrease of the total carbonation of the solution, as the solution is saturated at a lower CO_2(aq)_ concentration. Also, although the rates for the dissolution reactions are faster at higher temperatures, the equilibrium shifts towards the CO_2(aq)_ side. Hence, both the physicochemical and chemical equilibria are achieved faster. This effect of the process temperature on the different mechanisms and their equilibria is not discussed in similar studies considering the CFD analysis of the CO_2_-H_2_O system [13,17,31,32,36]. In the present study, the model was able to provide insight into the effect of temperature on the different mechanisms that constitute the process. The fact that the model was also validated for the three temperature values, using two separate experimental metrics, increases the added value of the study.

On the other hand, the CO_2_ inflow rate was found to affect the process dynamics through the transport phenomena. CO_2_ inflow increase leads to faster carbonation of the solution, as the mass transfer is enhanced between the two phases. Hence, the CO_2_ was also an influential parameter on the process dynamics, however with a different mechanism. Instead of affecting the rates and equilibria of the physicochemical mechanisms of the process, as in the case of temperature, the effect on the process dynamics is through the transport phenomena. Although the influence of the CO_2_ inflow can provide valuable information regarding process optimization, it has not been thoroughly discussed in relevant literature. In particular, the CFD models developed by Kim et al. studied the effect of initial concentration and tank height [31], or reactor design [13]. Hu et al. also studied the effect of different reactor designs [32], while Rigopoulos et al. did not perform parametric studies with their CFD model and focused mainly on precipitation kinetics [17].

The effect of the rotational speed was also studied. As mentioned in the previous section, the present model formulation cannot unravel the full effects of the rotational speed on the process, as its effect on bubble size cannot be investigated. However, the rotational speed was found to have some effect on the mixing and the distribution of the bubbles in the stirring tank, even under the current formulation. The effect of the rotational speed on the overall process dynamics was less significant than the effect of temperature and the CO_2_ inflow. Nevertheless, a different approach is needed for the overall assessment of the effects of the rotational speed on the overall process, which could be the subject of future model development.

## 4. Conclusions

In this work, a three-dimensional, Computational Fluid Dynamics model is developed for a carbonation precipitation tank reactor. The present work focuses on the carbonation aspect of the process and simulates the flow of CO_2_ bubbles, as well as CO_2_ dissolution and dissociation, within a mixing tank filled with water. The model was able to reveal the interplay between the mechanisms that dictate the process, while it was also validated using experimental measurements for two separate metrics (pH, CO_2_ outflow) for three different temperatures. This experimentally validated mechanistic insight is a novelty in the relevant scientific literature.

The analysis revealed that the bubble flow and interphase mass transfer, as well as CO_2_ dissolution and dissociation kinetics, all play a significant role in the carbonation process dynamics. The process temperature was found to be a very influential parameter on the process through its effect on the CO_2_ dissolution and dissociation kinetics, as well as their equilibria. The CO_2_ inflow rate was found to influence the process dynamics via the transport phenomena, specifically via the mass transfer between the bubbles and the liquid solution, while the impeller rotational speed influenced the bubble dispersion, leading to local effects and the non-uniform distribution of chemical species within the tank. The mechanistic analysis of the effect of the different process parameters, especially temperature and CO_2_ inflow rate, is also a novelty in the relevant scientific literature.

The present model is a phenomenological model, focused on the carbonation step, which will serve as a basis for the incorporation of the chemical species and reactions leading to precipitation, which will be the subject of future work. Although the present model can provide valuable insight into the overall process macroscopically, more local effects such as bubble breakage and coalescence are not taken into account. This means that although the model is robust and can be used for various reactor designs and process parameters, aspects of the process such as bubble size and dispersion cannot be adequately studied.

Besides the above limitations, the current computational approach can potentially pave the way towards an in-depth understanding of similar processes overall, allowing a knowledge-based approach towards the design and optimization of similar processes and reactors. Finally, the scale-up of lab-scale processes to pilot- or even industrial-scale reactors can be realized using such approaches that take into account the effect of the reactor geometry and operating conditions.

## Figures and Tables

**Table 1:** Parameter values used for the simulations.

Parameter	Description	Value	Reference
*Process conditions*			
T	Temperature	According to experiments	
Ν	Rotation rate	200 rpm	
J_CO2_	CO_2(g)_ Inflow rate	160 sccm	
*Liquid domain*			
D_CO2_	CO_2(aq)_ diffusion coefficient	2.35 × 10^−6^ ×exp(−2119/T)	[31,65]
H_CO2_	Henry constant for CO_2_-H_2_O	3.54 × 10^−7^×exp(2044/T)	[65]
μ_h2o_	H_2_O dynamic viscosity	0.001002 × 10^((A*(20−T)−B*(T−20)^2)/(T + C))^	[66]
ρ_h2o_	H_2_O density	1000 kg/m^3^	
ρ_co2_	CO_2(g)_ density	Ideal gas	
k_g_	Mass transfer coefficient	$\frac{2}{\sqrt{\pi }}\sqrt{Sc{Re}_{b}}\frac{D_{co2}}{d_{b}}$	Based on [40,42,54]
k_f_	Forward reaction rate constant	exp(1246.98 − 61,900/T − 183*ln(T))	[55,67]
k_b_	Backward reaction rate constant	exp(29.45 − 8018/T)	Fitted based on [68]
D_H_	Diffusion coefficient of dissolved H^+^ species	9.31 × 10^−9^	[69]
D_HCO3_	Diffusion coefficient of dissolved HCO_3_^−^ species	1.18 × 10^−9^	[69]
d_b_	Mean bubble diameter	2 mm (Fitted to experiments)	
*Gas domain*			
ρ	Gas density	Ideal gas	
μ	Gas viscosity	Kinetic gas theory	
D_co2-air_	Diffusion coefficient of CO_2(g)_ in air	Kinetic gas theory	

## Data Availability

The original contributions presented in this study are included in the article. Further inquiries can be directed to the corresponding author.

## Appendix A

### Appendix A.1. Grid Independence Study

In this section, the dependence of the model results on the computational mesh is presented. This is done by comparing the model results obtained while gradually refining the computational mesh used for the computations. The model results for the evolution of CO_2_ outflow, pH and CO_2(aq)_ as a function of time are presented in Figure A1, for the different computational meshes used.

The results of Figure A1 show that there is a small effect of the mesh size on the results regarding the CO_2_ outflow, CO_2(aq)_ concentration, and pH evolution as a function of time. A slightly faster increase of the CO_2_ outflow and CO_2(aq)_ concentration is observed for the two coarser meshes, while all meshes produce a very similar pH evolution.

In order to investigate the mesh dependency of the results in terms of spatial distribution within the domain, the CO_2(g)_ bubble volume fraction on the liquid surface is plotted in Figure A2, for the results obtained once the system has reached equilibrium. The results of Figure A2 are also plotted along a horizontal line on the liquid surface that crosses the region of the maximum volume fraction, in Figure A3.

In Figure A2 and Figure A3, the effect of the mesh size can be clearly seen. For the coarser mesh (Figure A2a), the spatial distribution of the bubble volume fraction shows that the CO_2_ are mainly situated between the baffles and the tank wall. As the mesh size increases, the bubble volume fraction shows a distribution between the CO_2_ inlet tube and the impeller shaft. Although the volume fraction profile and the values show a difference between Figure A2a–c, the results of Figure A2c,d are very similar, showing that the results were no longer dependent of the mesh, when the mesh size was increased from 243,940 elements to 378,707 elements.

Finally, in order to quantify the effect of the mesh, the total volume of the CO_2(g)_ bubbles within the stirred tank reactor are also plotted as a function of the mesh size in Figure A4. Furthermore, the deviation from the value obtained using the finer mesh is also plotted. As can be seen from the results of Figure A4, the models developed using a coarser mesh led to an underestimation of the total CO_2(g)_ bubbles volume within the tank, with the deviation decreasing as the mesh size increased. Models developed using a computational mesh consisting of more than 243,940 elements showed a small deviation of less than 1% with mesh size increase.

The analysis of the mesh dependency study presented in Figure A1, Figure A2, Figure A3 and Figure A4 leads to the conclusion that the selected mesh for further analysis of the carbonation process is the mesh of 243,940 elements, as further increase of the mesh size did not lead to considerable differences of the model results.
